# Feasibility of Workplace Health Promotion for Restaurant Workers, Seattle, 2012

**DOI:** 10.5888/pcd12.150093

**Published:** 2015-10-08

**Authors:** Claire L. Allen, Kristen Hammerback, Jeffrey R. Harris, Peggy A. Hannon, Amanda T. Parrish

**Affiliations:** Author Affiliations: Kristen Hammerback, Jeffrey R. Harris, Peggy A. Hannon, Amanda T. Parrish, School of Public Health, University of Washington, Seattle, Washington.

## Abstract

**Introduction:**

Restaurant workers are a large population at high risk for tobacco use, physical inactivity, and influenza. They are difficult to reach with health care interventions and may be more accessible through workplaces, yet few studies have explored the feasibility of workplace health promotion in this population. This study sought to identify barriers and facilitators to promotion of tobacco cessation, physical activity, and influenza vaccination in restaurants.

**Methods:**

Moderators conducted 7 focus groups, 3 with restaurant owners and managers, 2 with English-speaking workers, and 2 with Spanish-speaking workers. All groups were recorded, and recordings were transcribed and uploaded to qualitative-analysis software. Two researchers coded each transcript independently and analyzed codes and quotations for common themes.

**Results:**

Seventy people from the restaurant industry participated. Barriers to workplace health promotion included smoking-break customs, little interest in physical activity outside of work, and misinformation about influenza vaccinations. Facilitators included creating and enforcing equitable break policies and offering free, on-site influenza vaccinations. Spanish-speakers were particularly amenable to vaccination, despite their perceptions of low levels of management support for health promotion overall. Owners required a strong business case to consider investing in long-term prevention for their employees.

**Conclusion:**

Tobacco cessation and influenza vaccinations are opportunities for health promotion among restaurant workers, whereas physical activity interventions face greater challenges. Promotion of equitable breaks, limited smoking-break policies, and free, on-site influenza vaccinations could improve health for restaurant workers, who often do not have health insurance. Workplace interventions may be particularly important for Hispanic workers who have additional access barriers.

## Introduction

The restaurant industry employs a large population of workers who earn low wages and have few health benefits. Restaurants are the second-largest private-sector employer in the United States, employing more than 13.5 million people (1). In 2013, the average annual wage for restaurant workers was $18,180, assuming year-round, full-time work, compared with $46,440 among all US occupations (2). Only 14% of restaurant workers have employer-provided health insurance, making restaurant workers difficult to reach through traditional health care interventions (3).

Restaurant workers are at high risk for obesity, smoking, and influenza. A Washington State study of obesity showed that, similar to the average rate among all Washington employees, only 37% of food workers meet minimum physical activity recommendations (4). The food service industry has the highest smoking prevalence (30%), comparable only to construction and extraction industries (5). Furthermore, restaurant workers are at greater risk for influenza. One study among restaurant workers found that only 26% reported receiving influenza vaccinations (6). Although vaccination is the most effective method of preventing influenza, vaccination levels are only 29% for working adults ages 18 to 49 (7).

As workplaces, restaurants provide a venue to reach workers and improve their health. The Centers for Disease Control and Prevention’s *Guide to Community Preventive Services* (*Community Guide*) recommends evidence-based health promotion practices that are applicable to workplaces in the areas of tobacco cessation, physical activity promotion, and influenza vaccination (8–10), but there are no qualitative studies on the feasibility of these practices among restaurant workers. Research on restaurant workers focuses on alcohol use or stress and concentrates primarily on English-speaking workers (11,12). With an estimated 30% of unauthorized immigrants employed in the service sector, industry-specific studies are needed to gather data on the perspectives of both English and non-English-speaking workers (13,14).

The purpose of this study was to identify barriers and facilitators to workplace health promotion among restaurant workers at sit-down restaurants. Secondary aims were to compare perceptions between restaurant owners and workers and between English- and Spanish-speaking workers.

## Methods

We conducted 7 focus groups in July 2012 with workers at restaurants in King County, Washington. King County has a population of just over 2 million and is 63% non-Hispanic white and 9% Hispanic. King County has a strong public health infrastructure. It is recognized as a leader in eliminating health disparities, and smoking is prohibited indoors and within 25 feet of public places (15). We conducted 3 groups with restaurant owners and managers (hereafter referred to as owners), 2 with English-speaking workers, and 2 with Spanish-speaking workers. Focus groups are excellent tools for determining awareness, concerns, beliefs, and motivations among diverse populations. Because this was formative research aimed toward developing questions to be tested later, grounded theory was used to guide the study (16). The University of Washington Institutional Review Board determined that this study poses minimal risk and exempted it from further review. This manuscript follows the Consolidated Criteria for Reporting Qualitative Research guidelines (17).

### Recruitment eligibility

We used a convenience sample. A contracted commercial focus group facility (Consumer Opinion Services) recruited all participants through emails to names in databases of previous focus group participants and to industry lists supplied by a regional restaurant trade association. King County has approximately 2,000 sit-down restaurants, and about 3% of King County workers are employed by a sit-down restaurant (15,18). A sit-down restaurant was defined as a restaurant where food is served directly to the customers’ table. Participating owners owned, franchised, or managed at least 1 sit-down restaurant in King County, Washington. Participating workers spoke English or Spanish as their primary language and were employed full-time or part-time (minimum 10 hours per week) in a nonmanagement position at a sit-down restaurant in King County. We included Spanish-speaking workers because Hispanics constitute 25% of US restaurant workers (2). All participants were aged 21 or older. Restaurant owners and managers were offered $150 as an incentive to participate. Restaurant workers received $75.

### Focus group procedures

Focus groups were conducted at a focus-group facility in Seattle. One member of the research team (K.H.) moderated the English-speaking groups, and another member (C.A.), bilingual and trained in cultural competency, moderated the Spanish-speaking groups in Spanish. Interview questions were developed to inform a pilot study focused on effective workplace health promotion in a restaurant setting. The interview guides focused on motivations, barriers, and facilitators to offering and participating in workplace health promotion in the areas of tobacco cessation, physical activity, and influenza immunizations. The guide did not distinguish between on-the-job and leisure-time physical activity. At the end of each session, participants completed a brief survey covering demographics and health behaviors.

### Analysis

All focus groups were recorded. Audio recordings of the Spanish-speaking groups were transcribed in Spanish, then back-translated to English (GMR Transcription Services Inc). English-speaking groups were also transcribed (Proof Positive Transcriptions). Transcripts were imported into Atlas.ti (Scientific Software Development GmbH) and were coded and analyzed using thematic content analysis. Initial codes were categorized into higher-order codes reflecting emergent themes. One team member (C.A.) created the coding structure and 2 members (C.A. and K.H.) coded each transcript independently to identify themes, meeting frequently to ensure consistent interpretation of codes. We grouped data by topic area (tobacco cessation, physical activity, and influenza vaccination) and identified barriers and facilitators for each. We chose quotes from restaurant owners (RO), English-speaking workers (EW), and Spanish-speaking workers (SW) to represent central themes; quotes were included only when several participants stated similar ideas.

## Results

Seventy people (Table 1) participated in 7 focus groups (28 owners, 20 English-speaking workers, 22 Spanish-speaking workers). Most English-speaking workers were servers or bartenders (13/20), whereas most Spanish-speaking workers were dishwashers and food preparation workers (10/20), or cooks (6/20). Of the owners, 20 of 27 earned $41,000 or more annually, as did 8 of 19 of the English-speaking workers but none of the Spanish-speaking workers.

**Table 1:** Characteristics of 70 Restaurant Owners, Managers, and Workers Participating in Seven Focus Groups on Workplace Health Promotion, Seattle, Washington, 2012

Characteristic	Owners, n (N = 28)	English-Speaking Workers, n (N = 20)	Spanish-Speaking Workers, n (N = 22)
**Sex**			
Female	11	9	9
Male	17	11	13
**Age, y**			
18–24	0	5	5
25–34	6	8	5
35–44	9	3	3
45–54	6	3	5
≥55	7	1	4
**Education**			
<High school degree	0	0	6
High school degree or GED	1	4	9
Some college or vocational school	4	12	4
Degree from vocational school	3	2	0
Degree from college or university	17	2	1
Graduate or professional degree	3	0	1
Missing data	0	0	1
**Occupation**			
Bartender/server	0	13	3
Cook	0	5	6
Hosting/cashier	0	2	1
Dishwasher/preparation work	0	0	10
Owner/manager of franchise restaurant	11	0	0
Owner/manager of nonfranchise restaurant	17	0	0
Missing data	0	0	2
**Annual income, $**			
≤10,000	0	2	10
11,000–20,000	0	6	7
21,000–30,000	1	3	4
31,000–40,000	6	0	1
41,000–50,000	3	3	0
51,000–60,000	2	4	0
≥61,000	15	1	0
Missing data	1	1	0

### Tobacco cessation

All participants agreed that smoking among restaurant workers is common and spoke about smoking primarily in the context of smoking breaks. Owners perceived smoking as a disruption to productivity, English-speaking workers focused more on the social context of smoking, and Spanish-speaking workers discussed smoking as something done alone to relax from a stressful job: “I work at a very busy restaurant, and it’s stressful; and I relax when I smoke” (SW).

#### Barriers

The most frequently mentioned barrier to tobacco cessation in restaurants was that smoking breaks are the only way to get a work break in restaurants: “Other than if you go to the bathroom, you don’t get a break. You get to go to the bathroom or you get to have a cigarette” (EW).

Another barrier is that smoking is interwoven into the social culture of restaurant workers. Workers said they were prompted to smoke when coworkers smoked: “I mean, if she takes a smoking break, and if you like her, you’re going to smoke with her” (EW). Casual smoking while drinking alcohol was mentioned as a common restaurant habit.

Overwhelmingly, owners agreed that smoking is a private decision, and asking workers to stop smoking would be intrusive. Some English-speaking workers were concerned that tobacco cessation promotion efforts would single out individual smokers.

#### Facilitators

Owners were interested in reducing the number of smoking breaks. They expressed frustration about smokers taking frequent breaks during work hours and concern about tensions between smokers and nonsmokers caused by the perceived lack of break opportunities for nonsmokers: “The people who smoke are the ones who get the breaks. It’s irritating*”* (RO).

Although owners focused more on productivity issues caused by smoking breaks, all groups supported tobacco cessation promotion as an issue that affects the customer experience. The smell of smoke was a strong concern for all groups. Owners suggested limiting the hours during which workers are permitted to smoke while on the job and supported the idea of promoting a smoking cessation quitline to workers indirectly using posters or pamphlets. Owners thought directly speaking to employees about cessation was intrusive.

### Physical activity

All groups emphasized the physically rigorous nature of restaurant work, and participants thought that health issues other than physical activity were more relevant to the industry.

#### Barriers

Many participants mentioned feeling physically exhausted at the end of the workday and said that they did not have energy to exercise. Long workdays and varying schedules were also common barriers.

When asked to consider offering gym discounts, owners stated that because of high employee turnover, they did not have the luxury to invest in something that would provide only long-term health benefits. Owners and workers doubted that anyone would actually use gyms and said that workers would prefer health insurance or paid vacation: “They want health insurance. They don’t want a gym membership. They want health insurance” (RO).

Participants noted that the constant exposure to unhealthy food at work was more of an industry barrier to better health than lack of physical activity. Workers thought that restaurants promoting physical activity while selling unhealthy food sent an inconsistent message about the importance of employee health: “[Y]ou’re selling fried food and burgers and all that stuff and you’re trying to get me to go to the gym? I’d be like okay, well, do you want to make a better menu?” (EW).

#### Facilitators

Owners viewed social exercise (such as company sports teams) as a worthwhile investment because it could foster team-building, increase morale, and improve retention: “From a restaurant perspective I would want to see group activities that are team builders. That’s what I would be interested in investing in” (RO).

English-speaking workers emphasized that restaurants have a tight-knit social culture and thought company teams would be appealing. Spanish-speaking workers, however, did not think that they worked in a warm or collegial environment, and many felt like outsiders: “Most of [the people I work with] are Americans, some of them accept the Hispanic but some don’t. It’s difficult” (SW).

### Influenza vaccination

Participants said restaurants frequently require people to work when sick. Workers said that managers expect them to work regardless of their health: “I’ve been told to come to work when sick. . . . I just got through seeing a doctor and I had a note. ‘Well, we need you. We don’t have anyone to cover you.’ That happens all the time” (EW).

Owners said that workers often won’t mention when they’re sick because they can’t afford to miss work but also acknowledged that staffing shortages sometimes resulted in their inability to let workers stay home: “By the nature of the business, either they’re not telling you that they’re super sick because they want to work and they need to make money, or you’re under the gun and you need people to work. You’re like ‘take some Airborne and get your ass here’ ” (RO).

#### Barriers

A major barrier to influenza vaccinations for owners and English-speaking workers was their belief that influenza vaccinations cause illness: *“*I have friends and family who get the flu shot. Everybody says you get sick. I’m not going to put myself out there to get sick voluntarily!” (EW).

Owners and English-speaking workers believed that an influenza vaccination should be optional rather than mandatory and that making it mandatory would be inappropriate.

Some owners and English-speaking workers viewed influenza as something that young, healthy restaurant workers don’t need to worry about, whereas others said that getting influenza is inevitable when working with the public. For Spanish-speaking workers, the biggest barrier to influenza vaccination was being too busy working to get a vaccination.

#### Facilitators

All participants said the convenience of on-site influenza vaccination was the single most important facilitator to increasing vaccinations among restaurant workers. Many participants also emphasized the importance of offering vaccinations for free: “Obviously, I think if there was a person administering flu shots behind me right now, that’s the only way you would get staff to do it” (RO). “Some of us don’t have time to go to the doctor, or get a vaccine, because we’re at work. So if it’s accessible and it’s free, who wouldn’t do it?” (SW). 

Overall, owners were positive about potentially offering vaccinations and saw it as a benefit that both workers and customers would appreciate.

Spanish-speakers generally thought of influenza as more serious than English-speakers and were more willing to pay part of the cost or go out of their way for a vaccination: “If it’s for my health I’d pay it” (SW). Survey results also supported this idea (Table 2). Of Spanish-speakers, 21 of 22 said they would pay at least part of the cost for a vaccination, compared with 6 of 20 English-speaking workers. They were also more willing to come to work an hour early for a vaccination (20 of 22 vs 2 of 20), or walk 4 blocks to a pharmacy (18 of 21 vs 6 of 19).

**Table 2:** Self-Report of What Restaurant Workers (N = 44) Are Willing to Do to Obtain an Influenza Vaccination, Seattle, 2012

Survey Question	English-Speaking Workers (n = 20)	Spanish-Speaking Workers (n = 22)
**Come into work an hour early.**		
Yes	2	20
Yes, but only if paid for my time	2	2
No	16	0
**Walk to a pharmacy 4 blocks from work.**		
Yes	6	18
Yes, but only if paid for my time	5	1
No	8	2
Missing data	0	1
**Drive 15 minutes out of my way.**		
Yes	1	18
Yes, but only if paid for my time	4	1
No	14	2
Missing data	1	1
**Pay for a flu shot.**		
I would pay full price.	0	5
I would pay part of the price.	6	16
I would not pay anything.	14	1

### Owner–worker relationship

Two broad themes on the owner–worker relationship emerged. First, Spanish-speaking workers strongly asserted that owners did not value or care about Hispanic workers and therefore were unlikely to offer them support in health promotion: “The supervisor would say ‘it’s your problem, I don’t care if you smoke or not’” (SW). “The idea from a Hispanic is ‘work, work, work’ . . . the employer doesn’t even know your name” (SW). English-speaking workers did not express these views. Second, although owners were concerned about intrusion and privacy, workers indicated they would be receptive if health issues were communicated appropriately.

## Discussion

We found that owners will support health interventions only if clear benefits to their businesses exist. Tobacco cessation and influenza vaccinations were seen as opportunities for health promotion among restaurant workers; however, physical activity interventions did not align with owner and worker interests. 

Owners want to reduce tobacco use to increase productivity and improve the customer experience. Smoking breaks are a strong part of restaurant culture, and lack of enforcement of equitable breaks frustrates both owners and nonsmoking workers. Restaurant owners could limit smoking hours and enforce equitable breaks among all workers to reduce this tension. Creating smoking-break policies and providing information about tobacco quitlines could reduce tobacco use and is consistent with *Community Guide* recommendations when combined with incentives and competitions to motivate workers (19).

Increasing physical activity is a low priority for restaurants, because workers believe they are highly active during their workday. Owners and workers think encouraging physical activity in an industry that profits from unhealthy food is an inconsistent message. Therefore, any physical activity promotion in restaurants should be combined with healthy eating interventions. Although the *Community Guide* recommends combined strategies for workplaces, these are particularly difficult for restaurants given the nature of their industry (20). More research is needed on effective physical activity interventions for restaurant workers.

This study found that workers would be likely to get influenza vaccinations if restaurants offered them on-site. Offering them free is another facilitator, especially for English-speaking workers. Because myths about influenza vaccination (eg, causes sickness, only for the elderly) are common among English-speakers, providing on-site educational materials is crucial.

Owners support influenza vaccination because it could reduce absences from work and prevent staffing shortages. We found evidence of pressure from owners for workers to work when sick. However, it was also clear that missing a day of wages is often a hardship for low-wage workers. Paid-sick-leave policies may help workers meet their own and their families’ health needs; however, low-wage workers have little access to this benefit (21,22). Restaurants could institute paid sick leave with negligible financial impact (23). The paid-sick-leave laws recently enacted in Seattle and other cities may tilt the cost–benefit balance for owners in the direction of offering influenza vaccination to workers (24).

Findings on Spanish-speaking workers and influenza were somewhat surprising. Hispanic populations currently fall behind non-Hispanic white populations in influenza vaccination coverage by about 10%, and some studies suggest resistant attitudes may contribute to disparities (25,26). In our study, Hispanic workers expressed more willingness to get a vaccination than English-speaking workers, and resistant attitudes were almost nonexistent. Access was the greatest barrier. This finding provides qualitative support to studies with similar conclusions (25,27). Our study suggests that increasing access by offering on-site vaccinations at restaurants could help reduce disparities.

We found that Hispanic workers perceive that management does not value them. Language, cultural barriers, and discrimination may contribute to the divide between these groups. Worker privacy was a concern for owners, yet workers predominantly said health promotion efforts would be appropriate and appreciated. These findings are consistent with research on employer and worker perspectives in low-wage workplaces (28,29) and suggest that health promotion interventions in restaurants should facilitate communication by providing materials and information in the native languages of all workers and by increasing owner awareness of worker perceptions.

Limitations of this study include that it uses a small, convenience sample of sit-down restaurant workers from 1 US city. Because Seattle has a robust, progressive public health infrastructure, including a law prohibiting smoking in public places, the findings may not be generalizable to all restaurant workers. A strength of this study is that it is the first to describe perspectives of both English- and Spanish-speaking workers on workplace health promotion in restaurants. This is also the first qualitative study on feasibility of workplace health promotion among restaurant workers in the areas of tobacco cessation, physical activity, and influenza vaccination.

Tobacco cessation and influenza vaccination are areas of opportunity for health promotion in restaurants, while physical activity interventions face greater challenges. We applied these findings to a pilot study exploring on-site influenza vaccinations among restaurant workers in Seattle (30). We also presented findings to the Washington Restaurant Association, the Seattle Restaurant Alliance, and to local health jurisdictions across Washington State, with intentions of creating a partnership. Further dissemination opportunities include sharing results with the National Restaurant Association, public health practitioners, and health promotion vendors. Areas of future research opportunities include larger, quantitative surveys using robust sampling methods that explore differences in health promotion barriers and behaviors between Hispanic and non-Hispanic white workers; replication of this study among other industries, geographic areas, or types of restaurant settings; and exploring partnership opportunities between restaurants and health departments or other health promotion organizations.

## References

[1] 2014 Restaurant industry pocket factbook. Washington (DC): National Restaurant Association; 2014. http://www.restaurant.org. Accessed October 13, 2014.

[2] Food and beverage serving and related workers. Occupational outlook handbook 2014–15 edition. Washington (DC): Bureau of Labor Statistics, US Department of Labor; 2013. http://www.bls.gov/ooh/food-preparation-and-serving/food-and-beverage-serving-and-related-workers.htm. Accessed January 15, 2015.

[3] Sheirholtz H. Low Wages and few benefits mean many restaurant workers can’t make ends meet. Washington (DC): Economic Policy Institute; 2014. http://www.epi.org/publication/restaurant-workers/. Accessed January 5, 2015.

[4] Bonauto DK , Lu D , Fan ZJ . Obesity prevalence by occupation in Washington State, Behavioral Risk Factor Surveillance System. Prev Chronic Dis 2014;11:130219. Accessed November 25, 2014 10.5888/pcd11.130219 24406093 PMC3887052

[5] Lee DJ , Fleming LE , Arheart KL , LeBlanc WG , Caban AJ , Chung-Bridges K , Smoking rate trends in US occupational groups: the 1987 to 2004 National Health Interview Survey. J Occup Environ Med 2007;49(1):75–81. 10.1097/JOM.0b013e31802ec68c 17215716

[6] Parrish AT , Graves MC , Harris JR , Hannon PA , Hammerback K , Allen CL . Influenza vaccination status and attitudes among restaurant employees. J Public Health Manag Pract 2015;21(3):E10–5. 10.1097/PHH.0000000000000195 25504235

[7] McIntyre AF , Gonzalez-Feliciano AG , Santibanez TA , Bryan LN , Greby SM , Biggers BB , Flu vaccination coverage, United States, 2011–12 influenza season . Atlanta (GA): Centers for Disease Control and Prevention; 2013. http://www.cdc.gov/flu/professionals/vaccination/coverage_1112estimates.htm. Accessed November 25, 2014.

[8] Callinan JE , Clarke A , Doherty K , Kelleher C . Legislative smoking bans for reducing secondhand smoke exposure, smoking prevalence and tobacco consumption. Cochrane Database Syst Rev 2010;14(4):CD005992. 20393945 10.1002/14651858.CD005992.pub2

[9] Kahn EB , Ramsey LT , Brownson RC , Heath GW , Howze EH , Powell KE , The effectiveness of interventions to increase physical activity. A systematic review. Am J Prev Med 2002;22(4, Suppl):73–107. 10.1016/S0749-3797(02)00434-8 11985936

[10] Interventions to promote seasonal influenza vaccinations among non-health care workers. The guide to community preventive services. Atlanta (GA): Centers for Disease Control and Prevention; 2008. http://www.thecommunityguide.org/worksite/flunon-hcw.html. Accessed November 25, 2015.

[11] Moore RS , Cunradi CB , Duke MR , Ames GM . Dimensions of problem drinking among young adult restaurant workers. Am J Drug Alcohol Abuse 2009;35(5):329–33. 10.1080/00952990903075042 20180660 PMC2829730

[12] Petree RD , Broome KM , Bennett JB . Exploring and reducing stress in young restaurant workers: results of a randomized field trial. Am J Health Promot 2012;26(4):217–24. 10.4278/ajhp.091001-QUAN-321 22375571

[13] Gleeson S . Leveraging health capital at the workplace: an examination of health reporting behavior among Latino immigrant restaurant workers in the United States. Soc Sci Med 2012;75(12):2291–8. 10.1016/j.socscimed.2012.08.031 23017892

[14] Passel JS , Cohn D . A portrait of undocumented immigrants in the United States. Washington (DC): Pew Hispanic Center; 2009. http://www.pewhispanic.org/files/reports/107.pdf. Accessed November 25, 2014.

[15] King County, Washington (WA). http://www.city-data.com/county/King_County-WA.html. Accessed May 5, 2014.

[16] Charmaz K . Constructing grounded theory. Thousand Oaks (CA): Sage Publications Inc; 2014.

[17] Tong A , Sainsbury P , Craig J . Consolidated criteria for reporting qualitative research (COREQ): a 32-item checklist for interviews and focus groups. Int J Qual Health Care 2007;19(6):349–57. 10.1093/intqhc/mzm042 17872937

[18] US Census Bureau. Washington: 2002, 2002 economic census, accommodation and food services, geographic area series. 2005. http://www.census.gov/prod/ec02/ec0272awat.pdf. Accessed June 3, 2015.

[19] Leeks KD , Hopkins DP , Soler RE , Aten A , Chattopadhyay SK ; Task Force on Community Preventive Services. Worksite-based incentives and competitions to reduce tobacco use. A systematic review. Am J Prev Med 2010;38(2, Suppl):S263–74. 10.1016/j.amepre.2009.10.034 20117611

[20] Task Force on Community Preventive Services. A recommendation to improve employee weight status through worksite health promotion programs targeting nutrition, physical activity, or both. Am J Prev Med 2009;37(4):358–9. 10.1016/j.amepre.2009.07.004 19765508

[21] Clemans-Cope L , Perry CD , Kenney GM , Pelletier JE , Pantell MS . Access to and use of paid sick leave among low-income families with children. Pediatrics 2008;122(2):e480–6. 10.1542/peds.2007-3294 18676534

[22] Christensen K , Schneider B , Butler D . Families with school-age children. Future Child 2011;21(2):69–90. 10.1353/foc.2011.0016 22013629

[23] Appelbaum, E ; Milkman, R ; Elliott, L , Kroeger T. Good for business? Connecticut’s paid sick leave law. New York (NY): Center for Economic and Policy Research; 2014. http://www.cepr.net/documents/good-for-buisness-2014-02-21.pdf.

[24] Morales A , Martinez MM , Tasset-Tisseau A , Rey E , Baron-Papillon F , Follet A . Costs and benefits of influenza vaccination and work productivity in a Colombian company from the employer’s perspective. Value Health 2004;7(4):433–41. 10.1111/j.1524-4733.2004.74006.x 15449635

[25] Hebert PL , Frick KD , Kane RL , McBean AM . The causes of racial and ethnic differences in influenza vaccination rates among elderly Medicare beneficiaries. Health Serv Res 2005;40(2):517–37. 10.1111/j.1475-6773.2005.0e371.x 15762905 PMC1361154

[26] Lindley MC , Wortley PM , Winston CA , Bardenheier BH . The role of attitudes in understanding disparities in adult influenza vaccination. Am J Prev Med 2006;31(4):281–5. 10.1016/j.amepre.2006.06.025 16979451

[27] Centers for Disease Control and Prevention. Vaccination levels among Hispanics and non-Hispanic whites aged > or = 65 years — Los Angeles County, California, 1996. MMWR Morb Mortal Wkly Rep 1997;46(49):1165–8. 9408045

[28] Hannon PA , Hammerback K , Garson G , Harris JR , Sopher CJ . Stakeholder perspectives on workplace health promotion: a qualitative study of midsized employers in low-wage industries. Am J Health Promot 2012;27(2):103–10. 10.4278/ajhp.110204-QUAL-51 23113780 PMC4180021

[29] Hammerback K , Hannon PA , Harris JR , Clegg-Thorp C , Kohn M , Parrish A . Perspectives on workplace health promotion among employees in low-wage industries. Am J Health Promot 2015;29(6):384–92. 10.4278/ajhp.130924-QUAL-495 25162321 PMC5070972

[30] Graves MC , Harris JR , Hannon PA , Hammerback K , Parrish AT , Ahmed F , Promoting influenza vaccination to restaurant employees. Am J Health Promot 2015. 10.4278/ajhp.131216-ARB-643 26305606 PMC8281321

